# Remote Multi-Person Heart Rate Monitoring with Smart Speakers: Overcoming Separation Constraint

**DOI:** 10.3390/s24020382

**Published:** 2024-01-08

**Authors:** Thu Tran, Dong Ma, Rajesh Balan

**Affiliations:** School of Computing and Information Systems, Singapore Management University, Singapore 178902, Singapore; dongma@smu.edu.sg (D.M.); rajesh@smu.edu.sg (R.B.)

**Keywords:** heart rate monitoring, acoustic-based sensing, smart speakers, multi-person tracking, spatial localization, FMCW

## Abstract

Heart rate is a key vital sign that can be used to understand an individual’s health condition. Recently, remote sensing techniques, especially acoustic-based sensing, have received increasing attention for their ability to non-invasively detect heart rate via commercial mobile devices such as smartphones and smart speakers. However, due to signal interference, existing methods have primarily focused on monitoring a single user and required a large separation between them when monitoring multiple people. These limitations hinder many common use cases such as couples sharing the same bed or two or more people located in close proximity. In this paper, we present an approach that can minimize interference and thereby enable simultaneous heart rate monitoring of multiple individuals in close proximity using a commonly available smart speaker prototype. Our user study, conducted under various real-life scenarios, demonstrates the system’s accuracy in sensing two users’ heart rates when they are seated next to each other with a median error of 0.66 beats per minute (bpm). Moreover, the system can successfully monitor up to four people in close proximity.

## 1. Introduction

Heart rate is one of the key indicators used to evaluate individuals’ overall health. For example, changes in heart rate can be used to assess the state of the nervous system [1] and are used as a stress indicator [2]. In addition, rapid resting heart rate has been suggested as a risk factor for cardiovascular mortality [3], and heart rate dynamics are used to infer sleep stages, as these dynamics are more conspicuous during the later Rapid Eye Movement (REM) period [4]. Although traditional cardiac monitoring approaches such as electrocardiogram (ECG) can achieve high accuracy, these are contact-based approaches that are expensive, uncomfortable to wear for prolonged periods, and cumbersome to set up and use. Thus, they are not suitable for home monitoring or for patients with skin allergies or burn injuries, where skin-contact sensors are not feasible.

Recent advancements in remote sensing have suggested various ways to leverage radio frequency (RF) signals to monitor heart rate without any sensors or probes attached to the skin. These include Frequency Modulated Continuous Wave (FMCW) radar [5,6,7], WiFi [8,9], and millimeter wave [10,11]. In addition, acoustic signals [12,13,14,15] have been used to extract respiration and heart rate in a contactless manner. These approaches use a speaker to emit inaudible high-frequency waves, typically above 18 kHz, use microphones to capture the reflected signal after it bounces back from targets in the nearby areas, then analyze the reflected signal to extract valuable information such as the respiration rate and HR as well as the distance and the angle of the target relative to the transceiver.

**Figure 1:**
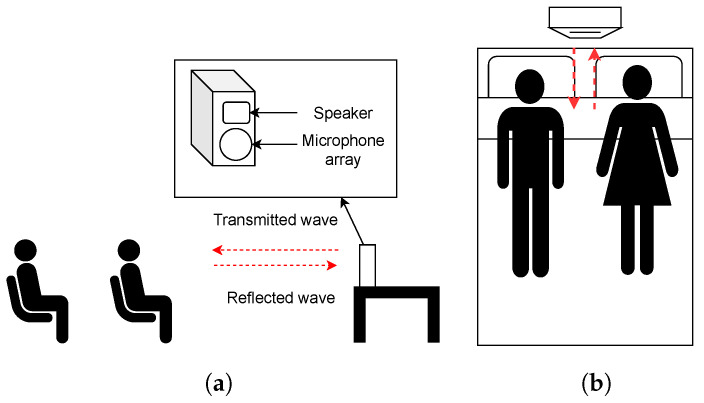
Practical scenarios of multi-person heart rate monitoring: (**a**) two people sitting in line and (**b**) Two people sharing a bed.

Even with extensive prior work in remote heart rate sensing, heart rate detection has focused mostly on single sensing. Only a few solutions target the sensing of multiple heart rates [6,16], and all of these only work under conditions that restrict their practical adoption. In particular, [6] requires the subjects to be 1 to 2 m separated away from each other when using RF signals; [12,16] require at least 40 to 50 cm separation as well as a 10° angular difference between each subject to achieve acceptable performance using acoustic signals. This leads to the infeasibility of tracking multiple people in real-life scenarios, such as sitting side by side or lying next to each other on the same bed, as shown in Figure 1.

In this paper, we aim to achieve multiple heart rate monitoring in such practical scenarios using a commodity smart speaker, the MiniDSP UMA-8-SP USB mic array [17], which has the same layout as the Amazon Echo Dot [18]. A smart speaker is considered as an appealing platform for contactless and acoustic-based heart rate monitoring for two key reasons. First, smart speakers have become increasingly prevalent in home environments, where they provide various voice-based services. Second, commercial smart speakers usually incorporate a microphone array design to deliver high-quality audio services. These microphone arrays offer high-resolution signals for active acoustic sensing that have been demonstrated to improve heart rate detection performance [13,19].

However, detecting and differentiating heart rates poses challenges when multiple people are in close proximity, as their acoustic reflection signals interfere with one another due to increased multipath interference. In this paper, we present an acoustic-based system that can extract multiple heart rates as well as their location with no separation requirement. To achieve this, we first separate users at different distances by processing the reflected FMCW signals. For each particular distance, we apply a Fast Fourier Transform (FFT) to extract the frequency of their corresponding heart rate. Then, we propose an algorithm to eliminate the interference and amplify the heart rate signal. Next, to further identify users’ spatial information, we leverage the microphone array equipped in the smart speaker and apply beamforming to obtain their azimuth angles.

To assess the effectiveness of our approach, we conducted a study approved by the Institutional Review Board (IRB) under various realistic conditions. Using data collected from ten couples, our system shows the possibility of accurate heart rate detection when two individuals are positioned in close proximity, with a median error of 0.66 beats per minute (bpm). Moreover, we demonstrate the scalability of our technique by successfully identifying the heart rates and locations of four individuals seated next to each other.

## 2. Related Work

### 2.1. Contact-Based Heart Rate Monitoring

Contact-based heart rate monitoring approaches typically require sensors attached to the human body during the measurement process. These sensors are usually ECG or photoplethysmography (PPG). ECG is the traditional and “gold standard” contact-based technique to measure cardiac signals [20,21]. When performing ECG, electrodes are attached to the patient’s skin at several spots, such as the chest or arms to record the electrical impulses of the heart when they travel across the electrodes. Both the strength and the timing of the pulses are monitored. Although ECG is highly accurate and widely used to diagnose heart-related diseases, this method is not suitable for home monitoring as it requires well-trained technicians and is done merely in clinical settings. The most common alternative monitors for home use are pulse meters or wrist bands [22,23]. They typically use PPG sensors, which contains a light source and a photodetector. The light source shines a green light onto the skin, and the changes in the light reflected back from the skin are monitored by the photodetector to extract associated heart pulses. Although this method is more convenient than ECG, it is still a contact-based approach, and is inapplicable to people with skin allergies or burn injuries.

### 2.2. RF-Based Heart Rate Monitoring

The past few years have witnessed growth in the number of studies that monitor vital signs, including respiration and heart rate, in a contactless manner through the use of RF waves. For example, well-studied RF-based approaches including broad-band FMCW radar [6,7], WiFi signals [8,9], RFID [24], and millimeter wave [11] have all shown accurate respiration rate detection even in the presence of more than one person. In addition to respiration sensing, [25,26,27] have demonstrated the possibility of heart rate detection using the these radar technologies. However, because heartbeat-induced chest displacement is subtle and always drowned out by respiration, only a few papers [6,11,28] have provided results for heart rate monitoring of more than one person; all of these papers require the subjects to be separated by a minimum of 1 to 2 m, making it infeasible to monitor two people sitting next to each other or sharing a bed. Furthermore, such RF-based systems require dedicated hardware, which generally leads to high cost.

### 2.3. Acoustic-Based Heart Rate Monitoring

Recently, the rise in popularity of acoustic-enabled mobile devices such as smartphones and smart speakers has introduced a potential alternative to RF-based heart rate measurement. Both RF and acoustic signals employ similar techniques to monitor respiration and heart rate. For instance, [29,30] developed an approach that utilizes the in-built speaker and microphone in smartphones and smart speakers to monitor the respiration rate in a single user. Similarly, [15] proposed a method for remotely monitoring heart rate using smartphones, while [13,14] suggested an approach using a smart speaker prototype with a single speaker–microphone pair. In order to expand the sensing range of these systems, [19] incorporated multiple microphones and a beamforming algorithm. However, due to signal interference, these studies remain unable to simultaneously monitor multiple people. Two recent studies [12,16] introduced novel beamforming algorithms that address this interference issue, enabling the monitoring of multiple heart rates; nevertheless, these algorithms require individuals to be separated by 40 to 50 cm and to be at least 10° apart. In our proposed approach, we first utilize a heatmap to identify users’ distances and heart rates, then apply beamforming on multiple microphones using the known distances and heart rates. This allows us to obtain the localization of the users while eliminating the separation requirement. A summary of the differences between our proposed approach and existing studies is presented in Table 1.

## 3. FMCW Background and Key Challenge

### 3.1. FMCW Background

**Figure 2:**
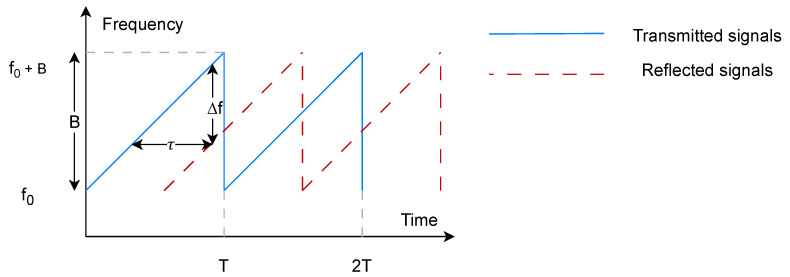
Transmitted and reflected FMCW signals.

In our proposed system, the speaker emits a sequence of FMCW chirps that are continuously modulated in frequency over a predefined time period. The reflected chirp is received by the microphone array, and the time difference between the transmitted and the reflected chirps indicates the range *R* between the transceiver and the object. For example, the blue line in Figure 2 shows the transmitted chirp with the frequency varying according to the sweep time *T*. The frequency of one single chirp is denoted as $f\left(t\right)=f_{0}+\frac{B}{T}t$, where $f_{0}$ and *B* indicate the start frequency and the bandwidth, respectively.

Therefore, a single chirp is represented as

(1) $$x\left(t\right)=cos\left(2\pi \int f(t)dt\right)=cos\left(2\pi (f_{0}t+\frac{Bt^{2}}{2T})\right).$$

The reflected chirp from a target to the receiver is the delayed version of the transmitted chirp, as shown in Figure 2, and is expressed as

(2) $$x^{\prime }\left(t\right)=\alpha cos\left(2\pi \left(f_{0}\left(t-\tau \right)+\frac{B{\left(t-\tau \right)}^{2}}{2T}\right)\right),$$

where α and τ refer to the signal amplitude attenuation factor and time delay, respectively. With a moving target, we have $\tau =\frac{2r(t)}{c}=\frac{2(R+vt)}{c}$, where *c* is the speed of sound 343 m/s, *v* is the speed of the moving subject, and r(t) is the distance of the moving subject by time. For a static object, $\tau =\frac{2R}{c}$; to compute the range *R*, we multiply the transmitted signal x(t) by the received signal $x^{\prime }\left(t\right)$. The mixed signal $x_{m}\left(t\right)$ is then represented as follows:

(3) $$\begin{array}{cc} x_{m}\left(t\right) & =x\left(t\right)\cdot x^{\prime }\left(t\right)\\ \; & =\frac{\alpha }{2}\left[cos\left(2\pi \left(f_{0}\tau -\frac{B(\tau^{2}-2t\tau )}{2T}\right)\right)+cos\left(2\pi \left(f_{0}\left(2t-\tau \right)+\frac{B(2t^{2}-2t\tau +\tau^{2})}{2T}\right)\right)\right]. \end{array}$$

The mixed signal consists of two terms. By taking the derivative of the phase by *t*, we have the frequency of the first term, which is a constant $\Delta f=\frac{B}{T}\tau =\frac{2BR}{cT}$. This implies that *every distance R maps to a specific frequency Δf.* The second term is a function of *t* with high frequency, and can be removed by a low-pass filter. In the end, after the multiplication and low pass filter, we have

(4) $$x_{mf}\left(t\right)=\frac{\alpha }{2}\cdot exp\left[j2\pi \left(f_{0}\tau -\frac{B(\tau^{2}-2t\tau )}{2T}\right)\right].$$

By transforming the frequency of $x_{mf}\left(t\right)$, we have

(5) $$R=\frac{cT}{2B}\Delta f.$$

Given a typical audio sensing bandwidth of B=5k Hz (usually from 18 kHz to 23 kHz), according to [30], the resolution of R is $\delta R\geq \frac{cT}{2B}\delta f=\frac{cT}{2B}\cdot \frac{1}{T}=\frac{343}{2\;\cdot \;5000}=3.43$ cm.

Although this resolution is sufficient to monitor centimeter-level human breathing, it is much lower than the heartbeat-induced chest displacement Δd, which is approximately 0.1–0.5 mm. Therefore, heart rate has been measured using phase-based methods [6,13,14,15]. It has been demonstrated that the minute chest displacement Δd can cause phase change in $x_{mf}\left(t\right)$ up to 18.9°. Specifically, $x_{mf}\left(t\right)$ can be expressed as follows.

(6) $$\begin{array}{cc} x_{mf}\left(t\right) & =\frac{\alpha }{2}\cdot exp\left[j(2\pi f_{0}\tau -\frac{\pi B\tau^{2}}{T}+\frac{2\pi t\tau B}{T})\right]\\ \; & \approx \frac{\alpha }{2}\cdot exp\left[j(2\pi f_{0}\tau +\frac{2\pi t\tau B}{T})\right]\\ \; & =\frac{\alpha }{2}\cdot exp\left[j\left(\frac{4\pi f_{0}}{c}r\left(t\right)+2\pi \Delta ft\right)\right]\\ \; & =\frac{\alpha }{2}\cdot exp\left[j\left(\frac{4\pi f_{0}}{c}\Delta d+2\pi \Delta ft\right)\right] \end{array}$$

As such, if the chest displacement caused by a heartbeat is 0.5 mm, the phase change is calculated as

(7) $$\frac{4\pi f_{0}}{c}\Delta d=\frac{4\pi \;\cdot \;18,000\;\cdot \;0.0005}{343}=0.105\pi =18.9^{\circ }.$$

Similar to prior works [6,29], in this paper we use distance to separate users. As each Δf indicates one *R*, we generate these frequency bins (which can be converted into distance bins) by applying FFT on $x_{mf}$, then analyzing the phase at each distance bin to determine the heart rate.

### 3.2. Key Challenge: Signal Interference

**Figure 3:**
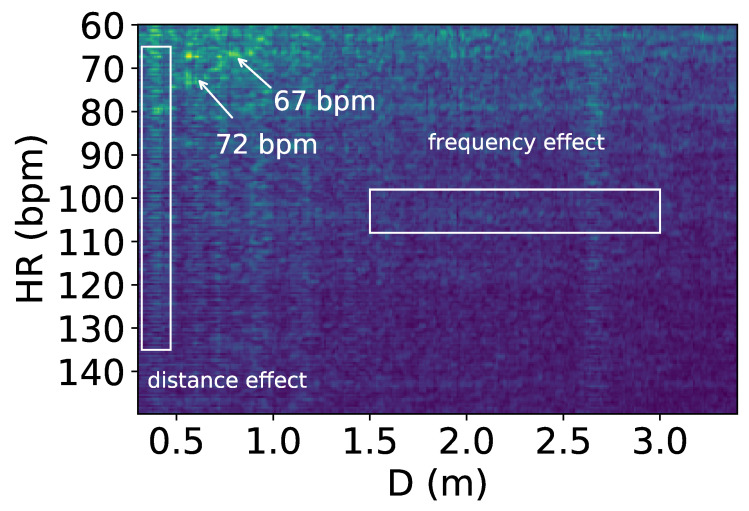
Heart rate–distance heatmap showing heart rates and interference from the reflected signals. The *x*-axis represents the distance *D* from the users to the device. In the figure, the two people with heart rates of 72 and 67 bpm located in front of the device cannot be distinguished visually from the heatmap.

The reflected signals at the microphone array undergo interference when more than one person is present. Figure 3 is a heart rate–distance heatmap generated from the reflected signals, showing the heart rates and distances of two people lying down next to each other; the device is placed above their heads, with brighter points indicating higher power. There are two heart rates of 72 and 67 bpm annotated in the figure; however, they are not visibly recognizable due to interference effects.

As shown in the figure, two types of effects are observed, which we name the *distance effect* and *frequency effect*. The distance effect happens when an object or person is located at a certain distance; it makes all the frequency power at that distance higher, as the frequency power is proportional to the power of the reflection signal. This signal is shown as bright columns in Figure 3). In the heatmap, it can be observed that this effect happens mainly at 0.5 to 1 m, which is the location of the two users.

The frequency effect, depicted as bright rows in Figure 3, is caused by multipath reflections bouncing off walls or furniture and arriving at the receiver with increased arrival time. This multipath effect has been well studied in the literature [31,32]. Despite the longer travel time, these reflections carry the same frequency information and are linearly correlated to the directly reflected wave, resulting in equivalent frequencies spanning a wide range of distances. Multipath signals have lower power than true reflections due to power loss over distance. We note that while these two effects can occur with a single person, they become more severe with multiple people, as interference is more likely. Our solution to this problem is proposed in Section 4.

## 4. System

### 4.1. System Overview

**Figure 4:**
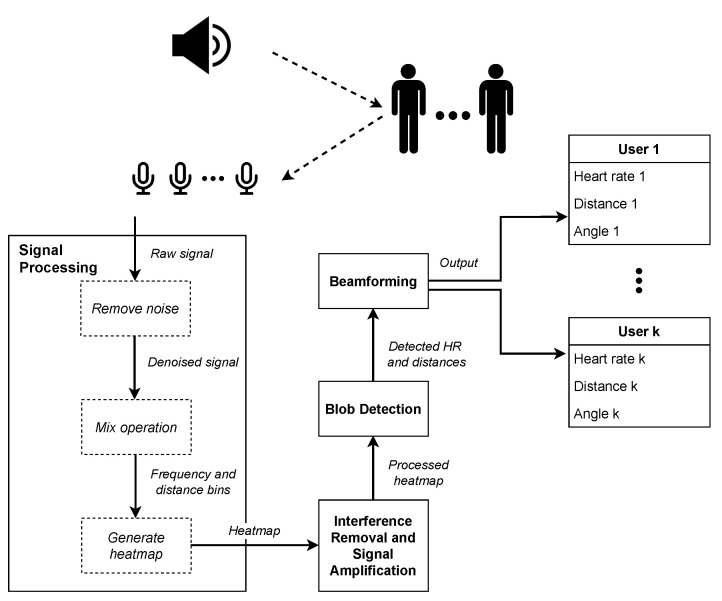
Overview of system for detecting the heart rates of *k* users.

Our proposed system uses a commercial speaker and circular seven-microphone array with the same microphone layout and sensitivity as a commodity Amazon Echo Dot [18] (MiniDSP UMA-8-SP USB mic array [17]) as an FMCW transceiver. The speaker emits FMCW signals and the microphone array captures the signal reflected by the user, allowing all heart rates, distances, and angles of users to be identified. As shown in Figure 4, our system has four main modules:

- **Signal Processing:** This module processes raw reflected signals received by the microphones, removes noise and frequencies outside the range of $f_{0}$ and $f_{0}+B$, and performs a mix operation on each chirp to generate the heart rate–distance heatmap.
- **Interference Removal:** The generated heatmap produced in the previous step is prone to distance and frequency interference effects. This interference is canceled through a two-step algorithm in order to highlight the heart rate signals.
- **Blob Detection:** Next, users’ heart rates and distances are detected by applying a blob detection algorithm to the heatmap.
- **Beamforming:** Finally, beamforming is applied to detect each user’s azimuth angle based on their distance and heart rate.

### 4.2. Signal Processing

**Remove Noise:** The raw signal received by each microphone is filtered using a bandpass filter between $f_{0}$ to $f_{0}+B$ to remove ambient noise such as human laughter and music, which are far lower than the operating FMCW frequency $f_{0}\geq 18k$ Hz [33], as such noise has little impact on the system.

**Mix Operation:** When the raw signal has been processed, a mix operation is applied to each received chirp by multiplying them by the transmitted chirp (Equation (3)). As explained in Section 3, Δf is proportional to the distance of the target and is obtained by performing FFT on one chirp. The frequency bins after FFT are converted into distance bins using Equation (5).

In the FFT, the frequency resolution is $\frac{F_{s}}{N}$, where $F_{s}$ is the sampling rate of the signal and *N* is the number of datapoints. Our system employs a sampling rate of $F_{s}=48k$ Hz and a chirp length of T=0.04 s. Therefore, the frequency resolution is $\frac{F_{s}}{T\;\cdot \;F_{s}}=\frac{48,000}{0.04\;\cdot \;48,000}=25$ Hz, which is converted to a *distance resolution* of 3.43 cm that is sufficient to differentiate users even when they are next to each other.

**Figure 5:**
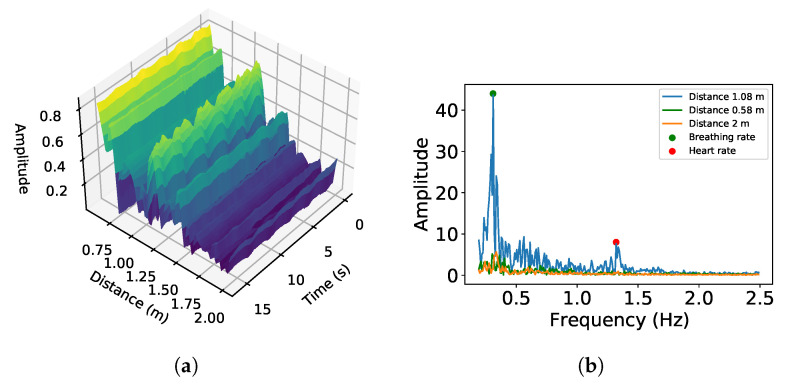
Amplitude changes and frequency domain of distances: (**a**) FFT amplitude changes by distance and (**b**) breathing and heart rate obtained by applying FFT at 1.08 m.

**Generate Heatmap:** The heart rate can be extracted using the phase changes of each distance bin over time. More particularly, we consider all distance bins within the device’s working range to find the bin that contains the heartbeat signal. To illustrate this, we collected data with a subject located 1.08 m away from the speaker. Figure 5a shows the amplitude changes of the distance bins ranging from 0.58 m to 2 m. It can be observed that the signal at 1.08 m shows a periodic pattern from the user’s breathing and heartbeats, while no vital signs can be seen at 0.58 m and 2 m. Figure 5b plots the frequencies for the 0.58 m, 1.08 m, and 2 m distance bins. These figures show that the bin with the highest amplitude, 0.58 m, is not guaranteed to contain heartbeat signals. This is because the distance effect (as the multipath) can cause the reflected signal to be stronger than the direct reflected signal. This phenomenon has been investigated in prior work [31,32].

Because we need to analyse the phase change of each distance bin, a chirp length of T=0.04 s leads to a $F_{sh}=\frac{1}{0.04}=25$ Hz sampling rate for the heart rate signal. Therefore, the frequency resolution of this FFT for heart rate is computed as $\frac{F_{sh}}{N}=\frac{25}{25\;\cdot \;60}=\frac{1}{60}\approx$ 0.0167 Hz, where 60 (s) is the window length of the signal to which FFT is applied. This frequency resolution is equivalent to 0.0167·60=1 bpm. To enhance this resolution we zero-pad the signal with 4096 samples prior to the FFT. This interpolation can yield a higher display resolution, in this case, $F_{sh}=\frac{25}{25\;\cdot \;60\;+\;4096}$≈ 0.004 Hz ≈ 0.3 bpm. We note that while this approach does not truly improve the frequency resolution, it provides a smoother FFT output, which allows peak-picking algorithms to achieve better accuracy.

Finally, this module produces a heart rate–distance heatmap. If more than one microphone is used, the heatmaps from each microphone are stacked into a single map by averaging their amplitude.

### 4.3. Interference Removal and Heart Rate Signal Amplification

The stacked heart rate–distance heatmap generated by the last step is affected by noise from the distance and frequency effects (see Section 3.2).

To remove these unwanted effects, we apply L1-normalization, a normalization technique that modifies the dataset values to ensure that the sum of the absolute values in each row always adds up to 1, to all the rows (i.e., heart rates) and then all the columns (i.e., distances). L1-normalization balances out those cases when entire columns or rows have similar amplitude while preserving the relative ratio of rows and columns that contain heartbeat signals. Next, we apply Gaussian smoothing to the heatmap to highlight the heart rates and remove noise. The algorithm is described in Algorithm 1.
**Algorithm 1:** Remove interference and amplify heart rate signals**Input**: Heatmap S, with n rows and m columns**Output**: Interference-free and amplified heatmap S
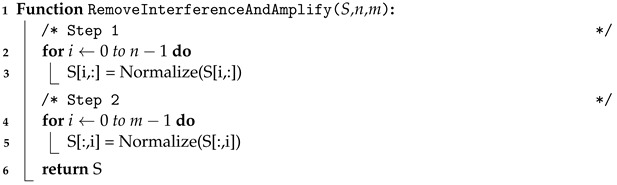


**Figure 6:**
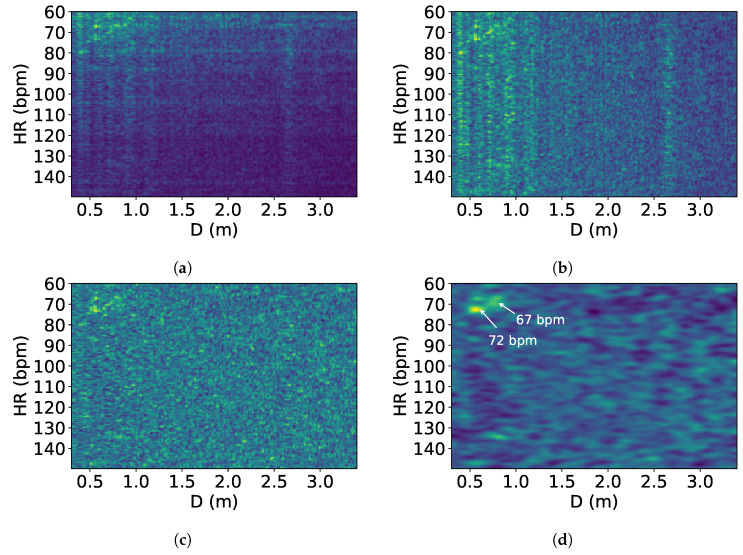
Interference removal: (**a**) original heatmap S; (**b**) heatmap S after step 1; (**c**) heatmap S after step 2; (**d**) smoothed heatmap S.

Figure 6b,c shows these steps to remove the effects, while Figure 6d shows the Gaussian smoothing used to highlight the heart rates.

### 4.4. Blob Detection

**Figure 7:**
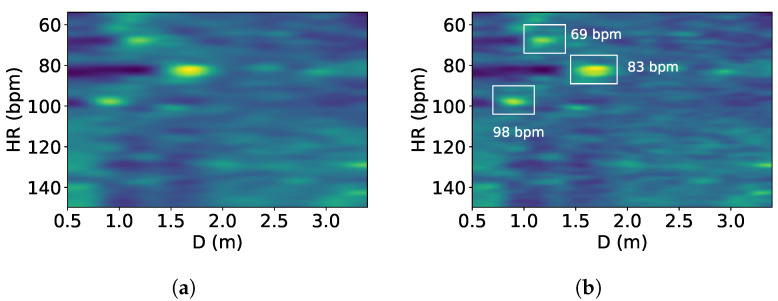
Three heart rates of 69 bpm, 83 bpm, and 98 bpm: (**a**) heatmap with three people and (**b**) the three brightest blobs.

A blob is a set of adjacent pixels that share common traits such as brightness or color. Because people can occupy many points in both distance and frequency, we detect the top brightest blobs in the heatmap instead of the top highest peaks. In this module, the input image is the heatmap from the previous step and the blobs to be detected are the elliptical bright spots on the image’s dark background that indicate users’ distances and heart rates. We apply the Laplacian of Gaussian (LoG) [34] as the primary blob detection method. With the blobs detected, we find the top *k* brightest blobs by calculating each blob’s mean value in the frequency range of 0.8 Hz to 2.5 Hz, with *k* being the number of users. Figure 7 shows detected distances and heart rates when there are three people in front of the device.

### 4.5. Beamforming

**Figure 8:**
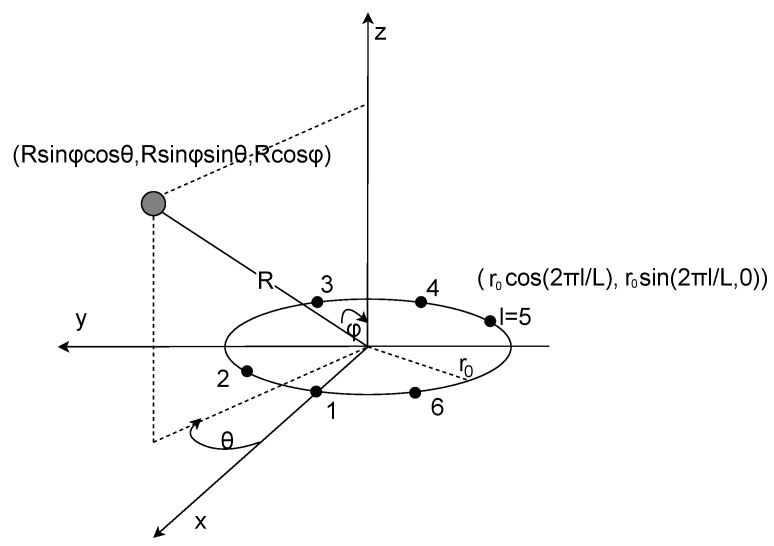
Source and circular microphone array with L = 6.

**Figure 9:**
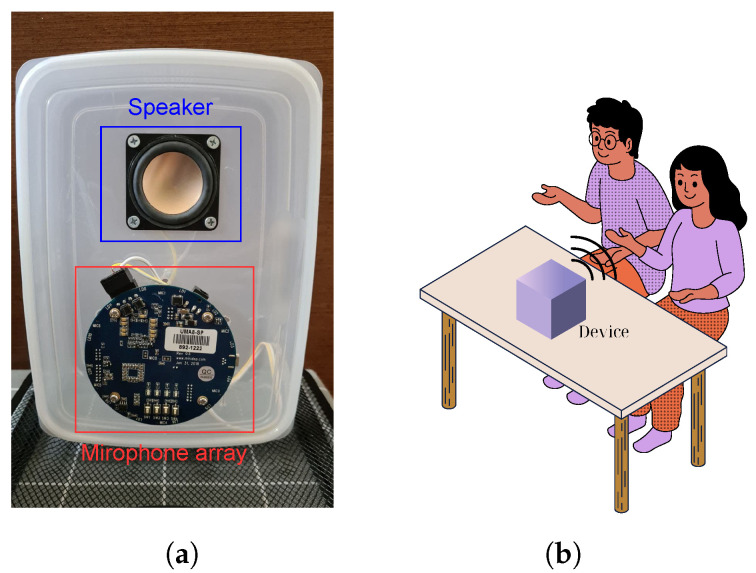
Device and example showing the experimental setup: (**a**) the device and (**b**) one of the experimental setups.

To further identify users’ locations in space, we apply digital beamforming on a circular microphone array [35] (Figure 8 and Figure 9a) to obtain their azimuth angles from the known heart rates and distances extracted in the last module. According to Equation (6), we can rewrite $x_{mf}$ using the *n*th distance bin and *i*th chirp:

$$x_{mf}\left(i,n\right)=\frac{\alpha }{2}\cdot exp\left[j\left(\frac{4\pi f_{0}}{c}r\left(f\left(n\right)+g\left(i\right)\right)+\frac{2\pi \tau B}{T}\left(f\left(n\right)+g\left(i\right)\right)\right)\right]$$

where *f* and *g* are the function that linearly converts the $n^{th}$ distance bin and $i^{th}$ chirp, respectively, into time *t* in seconds. Specifically, g(i)=T·(i−1),i≥1 and $f\left(n\right)=2\frac{ToDistance(n)}{c},n\geq 1$, where ToDistance(n) is a function to convert distance bin *n* to distance in meters.

Based on the azimuth angle θ provided by the circular microphone array in Figure 8, we project the source onto the x–y plane to obtain $\varphi =\frac{\pi }{2}$ with channel *l*; then, $x_{mf}$ can be expressed as

(8) $$\begin{array}{c} x_{mf}\left(l,i,n\right)=\frac{\alpha }{2}\cdot exp\left[j\left(\frac{4\pi f_{0}}{c}r\left(f\left(n\right)+g\left(i\right)\right)+\frac{2\pi \tau B}{T}\left(f\left(n\right)+g\left(i\right)\right)+2\pi \frac{r_{0}\cdot cos\left(\theta -\Theta \left(l\right)\right)}{c}\right)\right] \end{array}$$

where $r_{0}$ is the radius of the circular array, θ is the azimuth angle of the target, and $\Theta \left(l\right)=\frac{2\pi }{L}l$ is the relative angle at microphone *l*. When the target is static, we have

(9) $$\begin{array}{c} x_{mf}\left(l,i,n\right)=\frac{\alpha }{2}\cdot exp\left[j\left(\frac{4\pi f_{0}}{c}R+\frac{2\pi \tau B}{T}\left(f\left(n\right)+g\left(i\right)\right)+2\pi \frac{r_{0}\cdot cos\left(\theta -\Theta \left(l\right)\right)}{c}\right)\right]. \end{array}$$

Because we obtained distances and heart rates in the last step, we can represent $x_{mf}\left(l,i,n\right)$ as $x_{n,l}\left(i\right)$ when fixing the distance and channel. To obtain the heartbeats across different angles for a given distance, beamforming is performed over L=6 microphones:

$$y_{n}\left(i,\theta \right)=S^{H}\left(\theta \right)X_{n}\left(i\right)+W\left(i\right)$$

where $S\left(\theta \right)=[s_{1}\left(\theta \right),...,s_{L}\left(\theta \right)]$ is the steering vector towards angle θ (with $s_{l}=exp\left(j2\pi \frac{r_{0}\;\cdot \;cos(\theta -\Theta \left(l\right)}{c}\right)$), $X_{n}\left(i\right)=\left[x_{n,1}\left(i\right),\ldots ,x_{n,L}\left(i\right)\right]$, and W(i) is the Gaussian white noise. In our implementation, *n* includes a range of distances covering all users and $y_{n}$ is the average of all values of *n*.

## 5. Results

In this section, we report the detailed performance of the proposed system in various realistic scenarios, including sitting, lying down with different postures, in the presence of ambience noise, and with more than two users.

### 5.1. Experimental Setup

**Figure 10:**
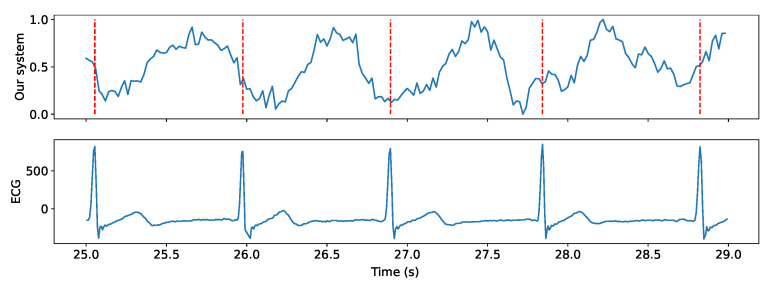
Extracted heartbeats of an individual located at 1 m and ground truth from ECG. The signal extracted from our system then undergoes FFT to obtain the heart rate in bpm.

We prototyped our system using an off-the-shelf seven-microphone circular array [17] connected to a speaker (PUI Audio AS05308AS-R), as shown in Figure 9a. This prototype has the same microphone layout and sensitivity as the widely used Amazon Echo Dot [18]. Table 2 provides information on all of the parameters used in our experiment. In addition, we employed Polar H10 ECG sensors [36] to collect heart rates for use as ground truth. Figure 10 illustrates the heartbeats extracted from our system and corresponding heartbeats collected by the ECG sensors. The metric used to evaluate our system was the heart rate (bpm), which we compared against heart rates captured by ECG sensors worn by participants. Figure 9b describes one of our experimental setups for two people sitting next to each other with no separation requirement.

To evaluate our system, we recruited ten couples (nine males and eleven females in the age range of 19 to 57 and with a median age of 26) to evaluate the impact of different parameters under various daily life scenarios. All experiments were approved by the IRB. We conducted further experiments with sets of three and four participants to verify that our proposed system can accurately monitor the heart rates of more than two people in close proximity.

### 5.2. Overall Performance

**Figure 11:**
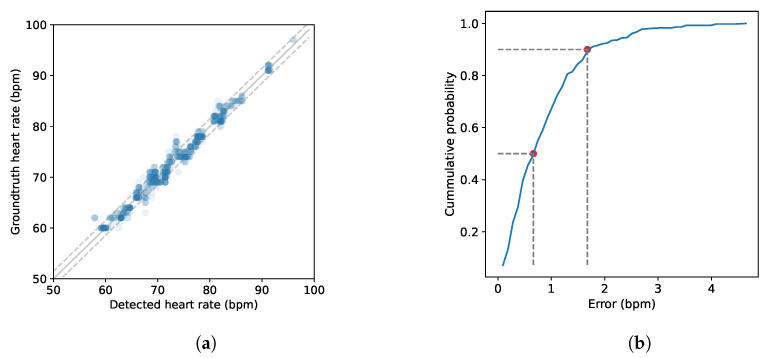
Overall evaluation of the system: (**a**) detected and ground truth heart rates in bpm and (**b**) cumulative distribution function of the error.

Initially, we assessed the system’s performance in everyday scenarios involving two individuals located in front of the device at an arbitrary distance from 0.5 to 1 m. Each recording session lasted for 2 min per couple, resulting in approximately 20 min of total recording time and a total of 1124 datapoints. As depicted in Figure 11a, the observed resting heart rates ranged from 57 to 96 bpm. The grey dashed lines represent two standard deviations. Additionally, Figure 11b illustrates that the achieved median error for heart rate estimation was 0.66 bpm and 1.67 bpm at the 90th percentile.

### 5.3. Impact of Distance

**Figure 12:**
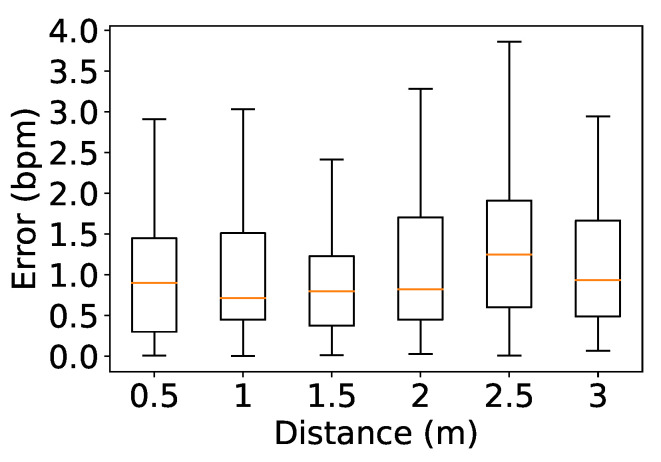
Impact of distance.

We asked ten couples to evaluate the impact of distance on the system’s performance when sitting next to each other in front of the device at various distances ranging from 0.5 to 3 m, with a step size of 0.5 m. The measurements were taken at each distance for 2 min while the couples are asked to remain stationary. Figure 12 shows median errors below 1 bpm when the distance was shorter than 2 m, with errors of 0.9, 0.71, 0.79, and 0.81 bpm at 0.5, 1, 1.5 and 2 m, respectively. Due to power loss, the median error increased slightly when the participants were located further from the device, with errors of 1.2 and 0.93 bpm at 2.5 and 3 m, respectively.

### 5.4. Impact of Angle

**Figure 13:**
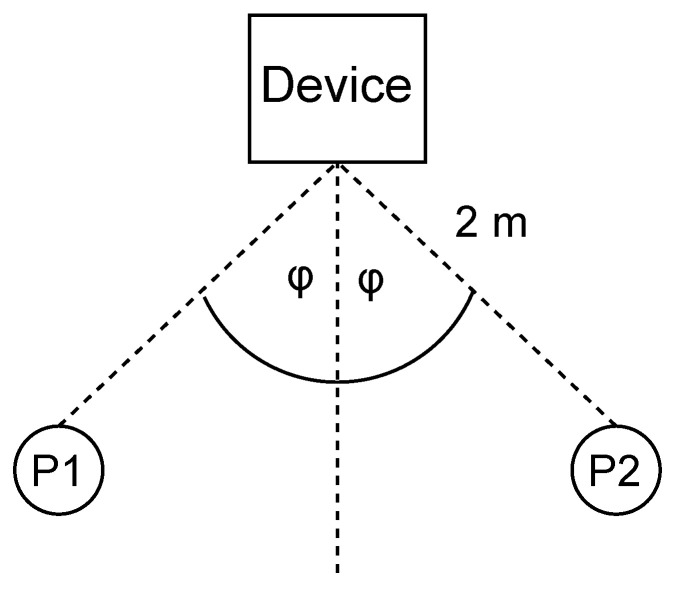
Results for users sitting at different angles; P1 and P2 refer to the two participants.

**Figure 14:**
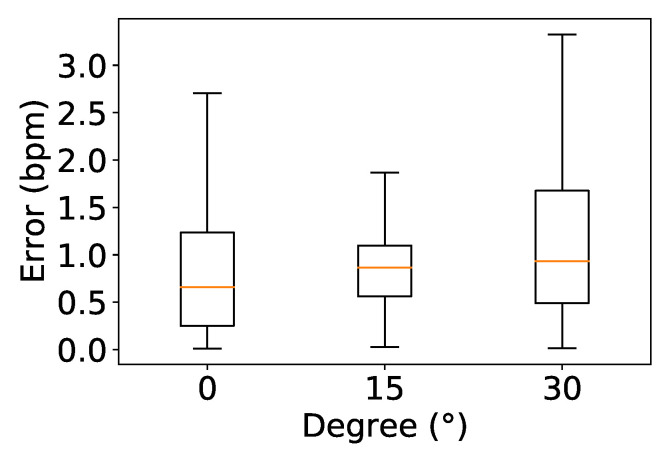
Impact of angle.

To assess the impact on the system of the angle between the users and the device, participants were asked to sit still at a fixed distance of 2 m with an angle φ changing from 0° to 15 ° and 30°, as shown in Figure 13. In Figure 14, it can be seen that the lowest median error when participants were seated at 0° was 0.65 bpm, while the highest median error was 0.93 bpm at 30°. We note that although the users were closer together when φ was lower, this did not decrease the accuracy very much. In fact, the error is mainly caused by the weak reflection when users are not directly facing the device, which leads to higher φ.

### 5.5. Impact of Ambient Noise

**Figure 15:**
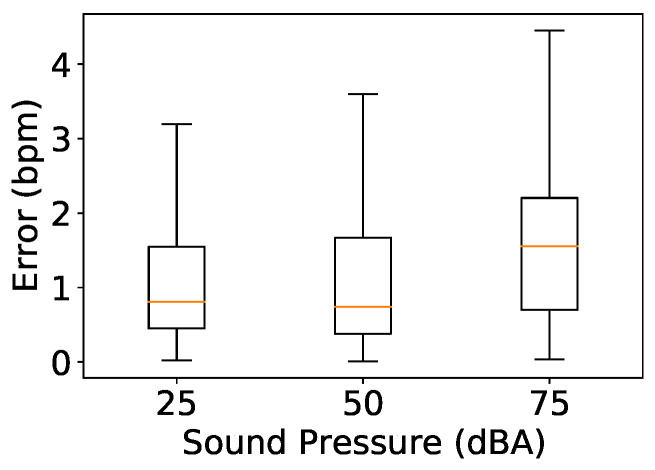
Impact of noise.

We investigated the impact of ambient noise by conducting the measurements in the presence of loud music. Each couple was asked to sit in front of the device at a distance of 2 m. The speaker was placed next to them while it played songs at 50 and 75 dB(A). These sound levels are comparable to normal conversation and road noise, respectively. Figure 15 shows the system performance under these two noise levels and when the average noise level when the room was quiet at 25 db(A). There was an increase in median error with higher sound pressure, especially when the sound pressure exceeded the sound pressure of the device itself, with the median error increasing to 1.5 bpm at 75 dB(A) compared to 0.8 bpm at 25 dB(A).

### 5.6. Lying Down with Different Postures

**Figure 16:**
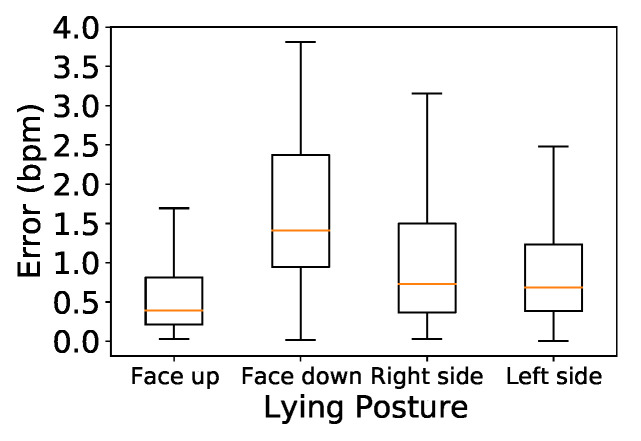
Impact of posture.

Different postures can lead to varying levels of accuracy. We conducted a user study involving couples lying down in the same bed in four common real-world postures: lying face-up, lying face-down, lying on the right side, and lying on the left side. To ensure that the participants could not block each others’ signals when performing different postures, we positioned the device 0.5 to 1 m away from their heads, similar to Figure 1b. The results are shown in Figure 16.

In Figure 16, it can be seen that the best accuracy of 0.38 bpm was achieved with both participants lying face-up, while the lowest (1.4 bpm) was found with both lying face-down. This is because the latter posture weakens the body displacement caused by the heart. Motion signals were slightly reduced for users lying on their right and left sides as compared to facing up, with the median error increasing to 0.72 and 0.68 bpm, respectively.

### 5.7. Lying Down with Blanket

**Figure 17:**
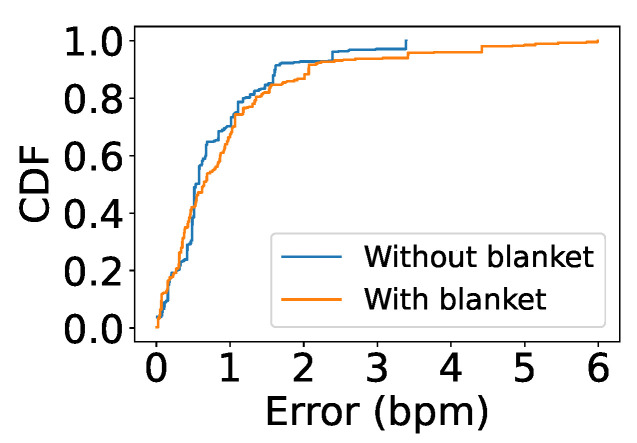
Impact of blanket.

We further evaluated our system with people lying face-up in bed covered by a blanket. As shown in Figure 17, the highest accuracy was achieved when both people were not covered by the blanket, with a median error of 0.53 bpm. Because thick cloth attenuates the signal, with the blanket there was a slight increase in the error to 0.64 bpm.

### 5.8. Impact of Movement

**Figure 18:**
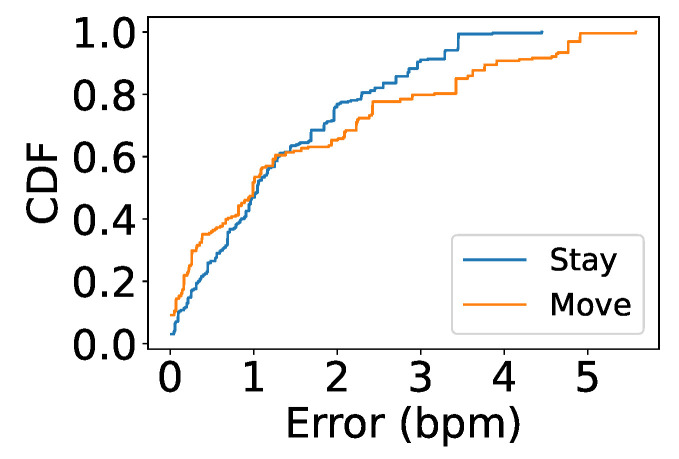
Impact of movement.

Instant body movements such as posture changes, talking, or phone swiping do not lead to the same rhythm as heartbeats. Hence, such movements should have little impact on measurement. To investigate the performance of our system when users are perform such sudden movements, we asked each couple to sit next to each other while they reading a text on their phones and scrolling down or up once in a while. Participants were asked to naturally change their posture if needed; in fact, such short-time motions sometimes cause noise in the same frequency range as the heart rate, as body movements lead to much larger phase changes compared to subtle heartbeat motions. To deal with this, we applied a heuristic method that assumes the correct heart rate will change very little over time while the noise will disappear in the subsequent time intervals. Despite a slight difference in the error distribution, Figure 18 shows comparable median errors between users sitting still and users performing sudden movements, with 0.9 and 1 bpm, respectively.

### 5.9. Impact of Number of Targets

**Figure 19:**
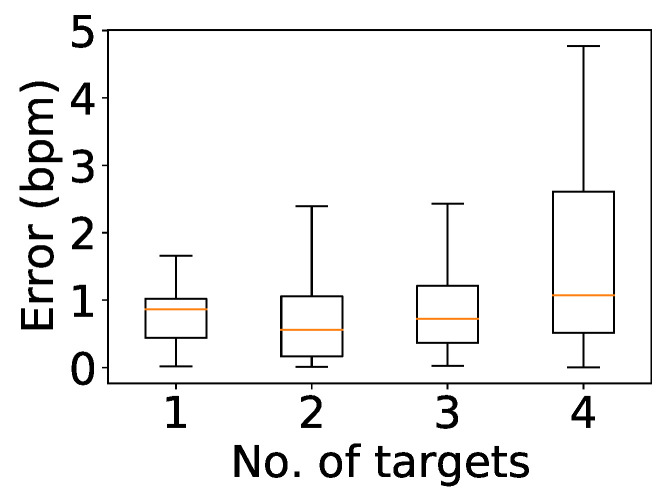
Impact of number of targets.

To evaluate our solution with up to four people, we asked groups of two, three, and four people to sit side by side for a 5-min trial at a distance of 2 m. As shown in Figure 19, the errors in all four cases matched the errors in Figure 12 for two people sitting at a distance of 2 m. Thus, it can be concluded that the accuracy of the system is not impacted even when increasing the number of users to four.

### 5.10. Impact of Number of Microphones

**Figure 20:**
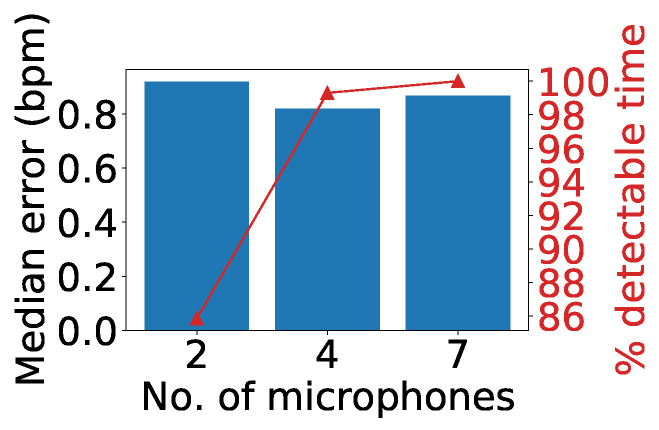
Impact of number of microphones.

To assess the system performance under different microphone arrays, we ran the collected data with two, four, and seven microphones in the array. For the array with two microphones, we selected the ones located at 120° and 240°; for the array with four microphones, we selected the ones located at 60°, 120°, 240°, and 300° (see Figure 8); and for the array with seven microphones, all of the microphones in the array were operating. Figure 20 shows that the median heart rate error was below 1 bpm in all three cases (0.91, 0.82, and 0.85 bpm for two, four, and seven microphones, respectively). However, regarding the detectable time, which refers to the time during which all users’ heart rates are visible in the heatmap, the two-microphone array had the lowest rate at 86%, compared to the seven-microphone one at 100%. This is because additional microphones improve the system’s ability to capture reflected signals from more directions.

### 5.11. Heart Rate Monitoring with Smartphone

**Figure 21:**
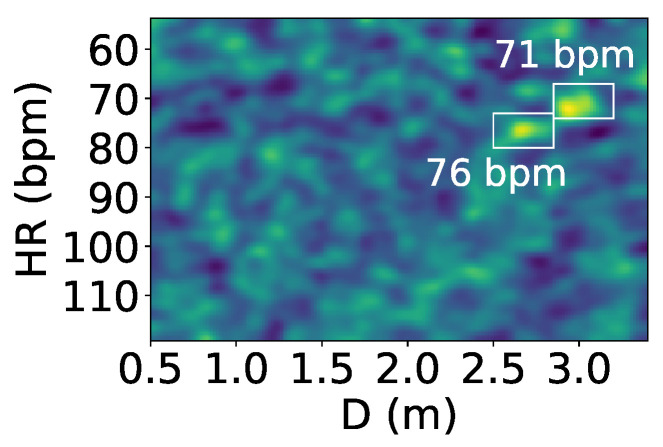
Heart rate detection by smartphone.

To examine the approach on another platform, we implemented our system on a Samsung Galaxy S20 Plus smartphone. Due to the phone’s design, it was only possible to use the single speaker–microphone pair at the bottom of the phone. Because only one microphone could be utilized, the angle information of the targets is not available. However, it remains possible to track the users by distance. We asked two volunteers to sit at distances of 2.7 m and 2.9 m in front of the smartphone. Figure 21 demonstrates that our system can achieve sensing of multiple heart rates at distances up to 3 m when deployed on a commercial smartphone with only one speaker–microphone pair; on the other hand, the sole existing smartphone-based approach [15] reports a maximum monitoring range of only 0.3 m.

## 6. Discussion

The following limitations may apply to our proposed method:

- **Prone to Rhythmic Movement:** Our approach can be susceptible to the impact of body movement, which is a known challenge for handling motion noise in acoustic-based methods. Because we assume that the user position falls within a frequency of 0.8 to 2.5 Hz (i.e., the normal heartbeat frequency range), any other modulation within this frequency range that does not originate from the human heart, although very unlikely, will confuse the system. As a result, although our system works well when the motion frequency is beyond the usual range of the heart rate (e.g., if the user shakes their head during measurement), it is suggested that users remain stationary during measurement to minimize possible noise that could fall within the heart rate frequency. In addition, we assumed that the device did not vibrate and that the background within the device’s working range contained no motion within the heartbeat frequency range. It is understood that voluntary or involuntary movement during signal acquisition typically reduces the fidelity of heart rate tracking [13,14].
- **Lack of Evaluation of Standing Postures**: While we conducted an extensive evaluation of our proposed system in various real-life scenarios, settings involving standing users were not included in our evaluation, as standing postures were not evaluated in any prior works in the literature. Therefore, we focused our evaluation on settings that were comparable with existing research. In fact, the standing setting is a challenge due to the difficulty of users maintaining stationary positions while in natural situations. For example, it is uncommon for an individual to remain completely still while standing for extended periods; a natural standing posture often involves walking or jogging. On the contrary, sitting or lying down naturally allows for more stationary positions. As a result, natural standing postures pose a great challenge in extracting subtle heartbeat signals, as we expect there to be significant motion noise. Consequently, we chose to leave the evaluation of different standing postures to future work.
- **Performance of Heart Rate Detection**: One assumption in our approach is that the normal heart rate falls within the range of 0.8 and 2.5 Hz, which is known as the normal heart rate range [37]. As such, any heart rate below 0.8 Hz may not be correctly detected, as the second harmonic of the respiration signal falls into this range and has significantly higher amplitude than the heartbeat.

## 7. Conclusions

In this paper, we present a remote approach to monitor the heart rates of multiple individuals using a commercial smart speaker with no separation requirement. Our proposed method removes interference and amplifies heart rates using a seven-microphone array on a smart speaker. This approach is able to separate heartbeat signals even when multiple users are sitting next to one another or lying down. Through our user study in various practical sitting and lying scenarios, the proposed approach is demonstrated to be highly accurate in these situations, with a median error of only 0.66 bpm. We believe that this approach can provide insightful inputs to other works, such as sleep stage classification, stress detection, and emotion classification.

## Figures and Tables

**Table 1:** Related works on heart rate monitoring using acoustic-based approaches. Each study’s median error is reported at its corresponding distance. In [13,14,15,19], the focus was on single users, while in [12,16] the authors monitored multiple users, for which the separation requirement is listed under *Separation*.

Study	Median Error (bpm)	Multiple Sensing	Separation	Distance (m)
[15]	0.6	No	Not applicable	0.3
[14]	0.75	No	Not applicable	0.2
[19]	0.6	No	Not applicable	0.6
[13]	1	No	Not applicable	0.4–0.6
[16]	0.8	Yes	40 cm and 10°	3
[12]	1.18	Yes	50 cm	3
Our approach	0.9	Yes	Not required	3

**Table 2:** Parameter settings for the experiments.

Parameter	Value
Chirp frequency	18 kHz to 23 kHz
Bandwidth	5 kHz
Chirp length	0.04 s
Maximum tracking distance	$\frac{cT}{4}=3.43$ m
Sampling rate	48 kHz
Sound pressure	45 dB(A) at 0.3 m

## Data Availability

The data are not publicly available due to privacy issues.
